# A nested case-control study on radiation dose-response for cardiac events in breast cancer patients in Germany

**DOI:** 10.1016/j.breast.2022.05.007

**Published:** 2022-06-09

**Authors:** Dan Baaken, Hiltrud Merzenich, Marcus Schmidt, Inga Bekes, Lukas Schwentner, Wolfgang Janni, Achim Wöckel, Manfred Mayr, Stephan Mose, Thomas Merz, Voica Ghilescu, Jona Renner, Detlef Bartkowiak, Thomas Wiegel, Maria Blettner, Heinz Schmidberger, Daniel Wollschläger

**Affiliations:** a University Medical Center of the Johannes Gutenberg-University Mainz, Institute of Medical Biostatistics, Epidemiology and Informatics, 55101, Mainz, Germany; b University Medical Center of the Johannes Gutenberg-University Mainz, Department of Obstetrics and Gynecology, 55101, Mainz, Germany; c University Hospital Ulm, Department of Gynecology and Obstetrics, 89075, Ulm, Germany; d University Hospital Würzburg, 97080, Würzburg, Germany; e Strahlentherapie Süd am Klinikum Kaufbeuren, 87600, Kaufbeuren, Germany; f Schwarzwald-Baar Klinikum, Klinik für Strahlentherapie und Radioonkologie, 78052, Villingen-Schwenningen, Germany; g Kliniken Landkreis Heidenheim gGmbH, Department of Radiation Oncology and Radiotherapy, 89522, Heidenheim, Germany; h University Hospital Ulm, Department of Radiation Oncology, 89081, Ulm, Germany; i University Medical Center of the Johannes Gutenberg-University Mainz, Department of Radiation Oncology and Radiation Therapy, 55101, Mainz, Germany

**Keywords:** Breast cancer, Radiotherapy, Cardiac mortality, Cardiac morbidity, Nested case-control study, Dose-response analysis

## Abstract

**Background:**

Previous studies with the majority of breast cancer (BC) patients treated up to 2000 provided evidence that radiation dose to the heart from radiotherapy (RT) was linearly associated with increasing risk for long-term cardiac disease. RT techniques changed substantially over time. This study aimed to investigate the dose-dependent cardiac risk in German BC patients treated with more contemporary RT.

**Methods:**

In a cohort of 11,982 BC patients diagnosed in 1998–2008, we identified 494 women treated with 3D-conformal RT who subsequently developed a cardiac event. Within a nested case-control approach, these cases were matched to 988 controls. Controls were patients without a cardiac event after RT until the index date of the corresponding case. Separate multivariable conditional logistic regression models were used to assess the association of radiation to the complete heart and to the left anterior heart wall (LAHW) with cardiac events.

**Results:**

Mean dose to the heart for cases with left-sided BC was 4.27 Gy and 1.64 Gy for cases with right-sided BC. For controls, corresponding values were 4.31 Gy and 1.66 Gy, respectively. The odds ratio (OR) per 1 Gy increase in dose to the complete heart was 0.99 (95% confidence interval (CI): 0.94–1.05, *P* = .72). The OR per 1 Gy increase in LAHW dose was 1.00 (95% CI: 0.98–1.01, *P* = .68).

**Conclusions:**

Contrary to previous studies, our study provided no evidence that radiation dose to the heart from 3D-conformal RT for BC patients treated between 1998 and 2008 was associated with risk of cardiac events.

## Highlights

• Dose-response analysis on cardiac late effects in irradiated breast cancer patients.
• Nested case-control study with individual retrospective heart dosimetry.
• Included 494 cases and 988 controls treated in 1998–2008 in Germany.
• No association for cardiac late effects of 3D-conformal radiotherapy observed.
• No association for the complete heart and left anterior heart wall observed.

## 1 Introduction

A cornerstone in breast cancer (BC) therapy, radiotherapy (RT) reduces local recurrence and BC-related mortality [1,2]. However, RT-induced risk for cardiac events is of clinical relevance to a growing number of long-term survivors [3]. Advances in RT, including improved treatment planning based on 3D-CT imaging [4], have reduced cardiac radiation dose [5]. Nevertheless, the heart remains exposed to ionizing radiation mainly depending on tumor laterality and individual anatomic risk factors [6]. On average, RT for left-sided BC is associated with higher radiation dose to the heart compared to right-sided BC [7]. Some studies have indicated an increased risk for radiation-induced cardiac effects based on tumor laterality [8,9], while others have not [10,11]. However, using laterality as a surrogate measure of exposure ignores large within-group heterogeneity of radiation doses in left-sided irradiated patients due to differences in individual anatomy and in radiation field geometry [7]. Therefore, dose-response analyses based on individual cardiac radiation dose estimates are better suited to quantify the radiation-induced cardiac risk. Darby et al. [12] showed a linear dose-response relationship between the mean dose to the whole heart (MHD) and risk of major coronary events in BC patients who received RT between 1958 and 2001 [12]. Of all included patients 76.3% were treated up to 1990, clearly before modern treatment planning came into widespread use. Furthermore, a systematic review and pooled analysis of 75 clinical trials with 40.781 patients mainly treated before 1990 and random assignment to RT of breast cancer patients identified an increased risk for cardiac mortality (rate ratio 1.30, 95% confidence interval (CI) 1.15–1.46) [13].

Using MHD to characterize cardiac radiation exposure has limitations since the heart is not a homogeneous organ. Damage to certain functional substructures, such as the coronary arteries, might be associated with characteristic late effects like ischemic heart disease [14]. Current studies raise the question whether dose to the left anterior descending (LAD) coronary artery is a better predictor for cardiac late effects compared to the MHD [15] because of the major role of the LAD in myocardial perfusion [14]. Furthermore, the heterogeneity of the cardiac dose distribution should be taken into account. Small regions of the heart in patients with left-tangential RT receive doses of ≥20 Gy even when MHD is low [5]. Using alternative dose metrics for risk assessment is also currently being investigated [16]. In a recent study [17], the volume of the left ventricle receiving ≥5 Gy (V5Gy) was shown to be a better predictor than MHD for acute coronary events in patients with BC. Jacobse and colleagues [18] identified an association between V5Gy for the complete heart and the rate of myocardial infarction. However, their results did not indicate that V5Gy was a better predictor compared to MHD.

Data about the risk of cardiac late effects in BC patients with contemporary RT based on individual heart dosimetry is still sparse. Here, we present the results of a nested case-control study to investigate a potential dose-response relationship between cardiac radiation exposure and cardiac late events in women diagnosed with BC between 1998 and 2008 in Germany, of whom >75% were treated after 2000.

## 2 Material and methods

### 2.1 Cohort population

The case-control study is nested within the ESCaRa cohort study (Epidemiological Study on Cardiac late effects and second malignancies after Radiotherapy in breast cancer patients) of 11,982 female BC patients [19]. Among them, 9057 (75.6%) were treated with 3D-conformal RT between 1998 and 2008 at Mainz University Medical Center's Department of Obstetrics and Gynaecology, Ulm University Hospital's Department of Gynaecology and Obestrics, or at one of 16 certified breast centers in the vicinity of Ulm. We included patients with histologically confirmed primary and locoregional BC, either an invasive carcinoma or a carcinoma in situ. We excluded patients with primary metastatic disease or bilateral BC. Details of the cohort were published previously [10,11]. In short, individual clinical data on disease characteristics, therapy, and comorbidities were obtained from patients' hospital records. These included date of birth and date of diagnosis, tumor laterality, TNM-stage, histological subtypes, grading, lymphatic and vascular invasion, hormonal status (estrogen and progesterone receptor), and treatment details.

An individual mortality follow-up was carried out to ascertain the vital status as of December 2012 via the compulsory municipal population registries of the patients’ last known residences. The underlying cause of death was coded according to the 10th revision of the International Classification of Disease (ICD), based on death certificates from local health authorities. Data on cardiac morbidity were assessed via a self-administered questionnaire in 2014 [11].

### 2.2 Nested case-control study

Cases and controls were selected among patients of the cohort who received 3D-conformal RT and had at least one year of follow-up. We applied 1:2 incidence-density sampling of controls with replacement [20]. Cases were defined as patients with RT who experienced a cardiac event later than the calendar year of their BC diagnosis. We distinguished two types of cardiac events: Either a self-reported incident event of cardiac morbidity from the questionnaire in 2014, or cardiac mortality until December 31st^,^ 2012. Cardiac morbidity included myocardial infarction, angina pectoris, congestive heart failure, dysrhythmias or valvular heart disease. Cardiac mortality was defined as the following causes of death: cardiac infarction (ICD I21–I23), chronic ischemic heart disease (ICD I25.0–25.9), acute ischemic heart disease (ICD I21.0–I24.9), congestive heart failure (ICD I50.0–I50.9), angina pectoris (ICD I20.0–I20.9), cardiac arrest (ICD I46), dysrhythmias and conduction disorder (ICD I44.0–I49.9), and vitium cordis (ICD I34.0–I37.9) [21].

Irradiated patients were eligible as controls if they had not experienced the type of cardiac event as the index case (cardiac morbidity or cardiac mortality) at the corresponding follow-up time of the event of the index case. Controls were matched based on their age at BC diagnosis (5-year age categories), year of BC diagnosis (5-year categories), the study center (Mainz, Ulm, partner clinic), and presence of a cardiac comorbidity at the time of the BC diagnosis. These baseline cardiac comorbidities were defined as having a New York Heart Association (NYHA) cardiac score ≥3 or a history of myocardial infarction, coronary heart disease, angina pectoris, dysrhythmias, vitium cordis, a stroke or pacemaker use. Information on baseline cardiac comorbidities was derived from clinical records, in particular from preoperative evaluation and preparation for anesthesia. When a relevant comorbidity was not mentioned in the documentation, it was coded as “No/unknown”. This label reflects that absence of a documented comorbidity might have resulted from a physician actively requesting the information that no comorbidity was present, or from failure of the physician to request that information.

### 2.3 Dosimetry

Independently from the nested case-control study, we selected a sample of 1353 patients from the ESCaRa cohort representative in terms of age and tumor laterality in order to retrospectively estimate individual heart dosimetry. Dosimetry details are described elsewhere [7]. Using 3D-conformal RT with tangential fields of 6 MV photons, the total radiation dose with regard to the planning target volume was typically 50 Gy. For breast-conserving treatment, an additional boost dose of 10 Gy was usually delivered to the tumor bed. RT could also include lymph node fields. In addition to the complete heart, several geometric surrogate volumes to functional anatomical heart structures were individually contoured, including the left anterior heart wall (LAHW). The LAHW contains the LAD, an important organ at risk that can be exposed to much higher doses compared to the complete heart [[22], [23], [24]]. The contouring was performed according to a heart atlas developed for retrospective epidemiological studies [25]. The volume-weighted mean dose (DMEAN) and the percentage volume of the structure receiving ≥5 Gy (V5Gy) were calculated for the complete heart and for the LAHW based on exported dose-volume histograms. The final sample of the nested case-control study consisted of 494 cases and 988 controls. For 91 cases and 182 corresponding controls, we were able to extract individually estimated DMEAN and V5Gy values for the complete heart and for the LAHW from the dosimetry sample. For 403 cases and 806 controls without individual dosimetry, doses were imputed as described below [22].

### 2.4 Statistical analysis

We used conditional multivariable logistic regression to calculate odds ratios (OR) and corresponding 95% Wald CI for estimated coefficients, conditioning on strata defined by the matching groups. The significance level was set at 5% without correction for multiple testing. We selected chemotherapy (yes/no), endocrine therapy (yes/no), and body mass index (BMI) (≥25.0/<25.0) as covariates based on a-priori theoretical considerations.

Multiple imputation using fully conditional specification methods [26,27] was used to deal with missing information on DMEAN and V5Gy for the complete heart and the LAHW, assuming data was missing at random. Doses were imputed using a previously validated dose prediction model based on age at breast cancer diagnosis, year of breast cancer diagnosis, study center, laterality and BMI [22]. We also used multiple imputation of missing values for BMI, chemotherapy, and endocrine therapy.

We evaluated potential nonlinearity of the dose-response association by adding a linear-quadratic term to our dose-response model for DMEAN of the complete heart. Goodness of fit was assessed by Akaike information criterion (AIC). In sensitivity analyses, we investigated a different dose metric (V5Gy) and a different functional substructure (LAHW). Furthermore, we carried out dose-response analyses for dose categories using quintiles from the observed distribution for DMEAN. For V5Gy, we used <10% as the reference category, and 10%–29% and ≥30% as further categories to ensure comparability with a previous study [18]. We also conducted sensitivity analyses restricted to only cases and their corresponding controls with available dose information from the dosimetry sample.

SAS 9.4 (SAS Institute North Carolina) was used for all analyses.

## 3 Results

### 3.1 Patient characteristics

Characteristics of the 494 cases and 988 controls included in the analysis are described in Table 1. The mean age at BC diagnosis was 63.14 for cases and 63.10 for controls. Most cases and controls were diagnosed with BC in 2004–2006 (43.12% and 44.64%, respectively). The cancer stage, individual risk factors at time of diagnosis (BMI, history of cardiac disease) and therapy-related characteristics were equally distributed between cases and controls (Table 1).

**Table 1:** Characteristics of cases and matched controls after breast cancer therapy in 1998–2008.

Characteristics	Cases		Controls	
	*N* = 494	100 (%^c^)	*N* = 988	100 (%^c^)
Year of breast cancer diagnosis				
1998–2000	62	12.55	129	13.06
2001–2003	94	19.03	182	18.42
2004–2006	213	43.12	441	44.64
2007–2008	125	25.30	236	23.89
Age at breast cancer diagnosis				
Mean	63.14	/	63.10	/
SD^a^	11.93	/	11.85	/
Laterality				
Left	264	53.44	540	54.66
Right	230	46.56	448	45.34
T-stage				
1	267	54.05	572	57.89
2	151	30.57	307	31.07
3	21	4.25	29	2.94
4	26	5.26	40	4.05
In situ	21	4.25	33	3.34
Unknown	8	1.62	7	0.71
N-stage				
0	311	62.96	586	59.31
1	111	22.47	248	25.10
2	34	6.88	77	7.79
3	15	3.04	45	4.55
X	23	4.66	32	3.24
BMI^b^				
<25.0	207	41.90	411	41.60
≥25.0	253	51.21	509	51.52
Unknown	34	6.88	68	6.88
History of cardiac disease^d^				
Yes	84	17.00	168	17.00
No/unknown	410	83.00	820	83.00
Chemotherapy				
Yes	215	43.52	398	40.28
No	276	55.87	576	58.30
Unknown	3	0.61	14	1.42
Endocrine therapy				
Yes	382	77.33	745	75.40
No	98	19.84	201	20.34
Unknown	14	2.83	42	4.25
Type of surgery				
None^e^	3	0.61	1	0.10
Breast conserving	425	86.23	843	85.32
Mastectomy	66	13.36	144	14.57
Unknown	0	0.00	0	0.00

a SD: Standard deviation.

b BMI: Body Mass Index.

c Percentages may not add up to a total of 100 due to rounding.

d History of cardiac disease at the time of breast cancer diagnosis, including history of cardiac infarction, coronary heart disease, angina pectoris, NYHA≥3, dysrhythmia, vitium cordis or use of a pacemaker.

e Patients who did not receive breast conserving surgery or mastectomy during their breast cancer therapy.

### 3.2 Radiotherapy-related characteristics

**Fig. 1:**
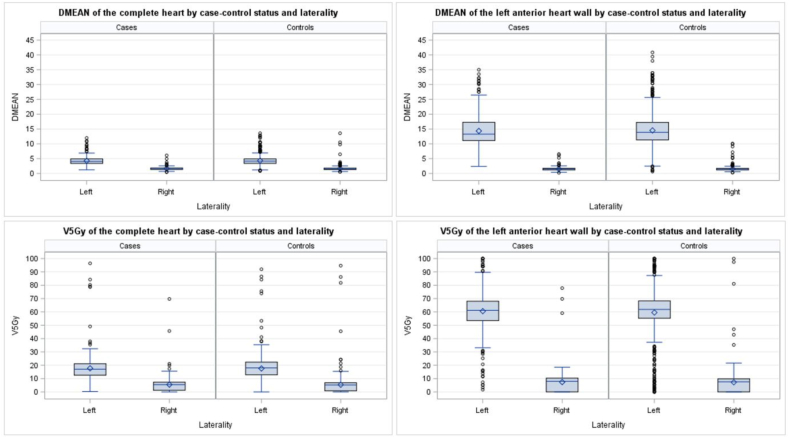
Boxplots of estimated mean heart dose and V5Gy of 494 cases and 988 matched controls after breast cancer therapy in 1998–2008 depending on case-control status and laterality for the complete heart and the left anterior heart wall based on dose predictions using multiple imputation.

Fig. 1 illustrates the distribution of estimated absorbed dose metrics DMEAN and V5Gy for the complete heart and for the LAHW. The average DMEAN ± standard deviation (SD) of the complete heart was in the same range for cases (left-sided = 4.27 ± 1.57 Gy, right-sided = 1.64 ± 0.60 Gy) and controls (left-sided = 4.31 ± 1.74 Gy, right-sided = 1.66 ± 0.94 Gy). Doses for left-sided RT were much higher compared to doses for right-sided RT. The same observations apply to the average DMEAN of the LAHW (Table 2).

**Table 2:** Mean, median and standard deviations for the complete heart and left anterior heart wall for different exposure metrics for 494 cases and 988 matched controls after breast cancer therapy in 1998–2008 stratified by laterality based on dose predictions using multiple imputation.

Structure	Metric	Laterality	Mean		Median		SD		Range	
			Cases	Controls	Cases	Controls	Cases	Controls	Cases	Controls
Complete heart	DMEAN [Gy]	Left	4.27	4.31	4.08	4.15	1.57	1.74	1.21–11.98	0.80–13.56
		Right	1.64	1.66	1.57	1.50	0.60	0.94	0.44–6.10	0.56–13.56
	V5Gy [Gy]	Left	17.71	17.61	17.05	18.02	11.68	10.13	0.28–96.45	0–91.99
		Right	5.47	5.37	5.44	5.18	6.29	7.96	0–69.74	0–94.69
Left anterior heart wall	DMEAN [Gy]	Left	14.34	14.53	13.26	13.87	6.12	6.35	2.37–34.99	0.65–40.85
		Right	1.53	1.55	1.44	1.39	0.70	0.91	0.23–6.51	0.30–10.09
	V5Gy [Gy]	Left	60.57	59.54	61.25	61.87	17.86	19.26	1.68–100	0–100
		Right	7.40	7.22	7.96	7.59	8.70	9.04	0–77.82	0–100

The supplementary information (Table A.1) presents patient characteristics of the 91 cases and 182 controls with observed individual dose from the dosimetry sample as well as the distribution of their doses (Table A.2 & Figure A.1). In general, the characteristics of cases and controls from the dosimetry sample (Table A.1) are in line with characteristics of those cases and controls of the complete group (Table 1). The predicted doses of the complete group correspond to the doses of the dosimetry sample, although they tend to be slightly higher.

### 3.3 Conditional logistic regression

We did not observe a linear dose-response relationship between DMEAN of the complete heart and risk for a cardiac event in the adjusted analyses (OR per 1 Gy increase = 0.99, 95% CI 0.94–1.05, *P* = .72). The same applies to the crude model. Analyses of dose categories did not reveal a monotonic trend with increasing dose categories, neither in crude nor in the adjusted analyses (Table 3). Adding a linear quadratic term to our dose-response model did not result in an improved model fit. Analyses of DMEAN of the LAHW did not reveal any dose-response relationship in the crude and adjusted model (OR per 1 Gy increase = 1.00, 95% CI 0.98–1.01, *P* = .68). The same holds for the categorical analyses (Table 3).

**Table 3:** Conditional logistic regression analyses of potential risk factors associated with cardiac morbidity and cardiac mortality of 494 cases and 988 matched controls after breast cancer therapy in 1998–2008.

Variables	Crude			Adjusted†		
	Odds Ratio	95% CI	*P*	Odds Ratio	95% CI	*P*
Dose groups for complete heart using DMEAN [Gy]						
1st quintile (<1.24 Gy)	1.00^a^			1.00^a^		
2nd quintile (≥1.24 Gy - <1.87 Gy)	1.00	0.63–1.58	.99	0.99	0.63–1.58	.98
3rd quintile (≥1.87 Gy - <2.84 Gy)	1.02	0.66–1.56	.93	1.01	0.66–1.55	.96
4th quintile (≥2.84 Gy - <4.60 Gy)	1.02	0.62–1.68	.94	1.01	0.61–1.67	.96
5th quintile (≥4.60 Gy)	1.00	0.64–1.55	.99	0.99	0.64–1.54	.97
Complete heart using DMEAN [Gy], continuous per 1 Gy	0.99	0.94–1.05	.73	0.99	0.94–1.05	.72
Dose groups for left anterior heart wall using DMEAN [Gy]						
1st quintile (<1.11 Gy)	1.00^a^			1.00^a^		
2nd quintile (≥1.11 Gy - <2.37 Gy)	1.00	0.64–1.55	.98	0.98	0.63–1.53	.94
3rd quintile (≥2.37 Gy - <7.38 Gy)	1.06	0.68–1.66	.80	1.05	0.67–1.66	.82
4th quintile (≥7.38 Gy - <15.12)	0.94	0.61–1.43	.76	0.94	0.61–1.42	.76
5th quintile (≥15.12 Gy)	0.94	0.62–1.42	.76	0.93	0.62–1.41	.74
Left anterior heart wall using DMEAN [Gy], continuous per 1 Gy	1.00	0.98–1.01	.68	1.00	0.98–1.01	.68
Chemotherapy						
No	1.00^a^			1.00^a^		
Yes	1.18	0.93–1.50	.18	1.20	0.94–1.54	.15
Endocrine therapy						
No	1.00^a^			1.00^a^		
Yes	1.05	0.80–1.39	.72	1.09	0.82–1.45	.61
BMI^b^						
<25.0	1.00^a^			1.00^a^		
≥25.0	0.99	0.78–1.24	.91	0.98	0.78–1.24	.89

† Radiation-related factors are adjusted for chemotherapy, endocrine therapy and BMI. Chemotherapy, endocrine therapy and BMI are adjusted for each other and DMEAN of the complete heart as continuous variable.

a Reference category.

b BMI: Body Mass Index.

No increase in risk for a cardiac event was observed in association with the proportion of the complete heart that received more than 5 Gy (Table 4). The proportion of the LAHW receiving more than 5 Gy was not a significant risk factor (OR per 1% increase = 1.00, 95% CI 0.996–1.004, *P* = .99). We observed a small but statistically non-significant increased OR of 1.06 (95% CI 0.67–1.67, *P* = .82) in the categorical analyses for the category 10%–29% of the LAHW receiving more than 5Gy compared to the reference category (<10%). Crude ORs were in line with the adjusted ORs (Table 4).

**Table 4:** Conditional logistic regression analyses of percentage of heart volume receiving >5 Gy and cardiac morbidity and cardiac mortality of 494 cases and 988 matched controls after breast cancer therapy in 1998–2008.

Variables	Crude			Adjusted^b^		
	Odds Ratio	95% CI	*P*	Odds Ratio	95% CI	*P*
Groups of percentage of the complete heart receiving >5 Gy [V5Gy]						
<10%	1.00^a^			1.00^a^		
10%–29%	1.01	0.76–1.35	.95	1.00	0.75–1.34	.99
≥30%	0.94	0.60–1.46	.78	0.94	0.60–1.47	.78
Percentage of the complete heart receiving >5 Gy [V5Gy], continuous per 1%	1.00	0.99–1.01	.96	1.00	0.99–1.01	.98
Groups of percentage of the left anterior heart wall receiving >5 Gy [V5Gy]						
<10%	1.00^a^			1.00^a^		
10%–29%	1.06	0.67–1.67	.80	1.06	0.67–1.67	.82
≥30%	1.02	0.79–1.31	.90	1.02	0.79–1.32	.88
Percentage of the left anterior heart wall receiving >5 Gy [V5Gy], continuous per 1%	1.00	0.996–1.004	.99	1.00	0.996–1.004	.99

a Reference category.

b Adjusted for chemotherapy, endocrine therapy and BMI.

Sensitivity analyses restricted to cases and their corresponding controls with available individual dose information did not alter the results of the main analysis (Table A.3 & Table A.4). No significant increased risk for cardiac events was observed, neither for the complete heart nor for the LAHW. This observation applies to both dose metrics, DMEAN and V5Gy.

## 4 Discussion

### 4.1 Main results

We assessed the risk for cardiac events after RT in female BC patients treated between 1998 and 2008 in Germany with a nested case-control analysis including 494 cases and 988 controls. We did not observe a significantly increased risk for cardiac events per Gy increase of DMEAN to the complete heart or LAHW in BC patients. Additional multivariable conditional logistic regression revealed no evidence for an increased risk for cardiac events associated with V5Gy for the complete heart or the LAHW.

### 4.2 Comparison to earlier studies

Our findings are generally supported by recent studies that used tumor laterality as a surrogate measure, showing no significantly increased risk for cardiac late effects for more recent treatment periods including 1998–2008 [19], 1999–2006 [28], 2000–2008 [29], 2000–2009 [30], 2001–2005 [31]. In addition, our results are in line with two recently published large clinical trials with long-term follow-up [32,33]. After a median follow-up of 15.7 years [32] and 34 years [33] no increased risk for cardiac death was observed comparing patients randomly assigned to lymph node radiation [32], or chestwall and regional lymph node radiation after mastectomy [33], respectively. However, studies based on individual dosimetry yield different results. Darby et al. [12] reported a linear increase for rates of major coronary events of 7.4% (95% CI 2.9–14.5, *P* < .001) per 1 Gy mean dose to the complete heart for patients treated between 1958 and 2001. In an update of the Danish part of Darby et al. an increased risk of 19% (95% CI 1%–63%, *P* = .02) per Gy of MHD for major coronary events was reported [34]. Jacobse et al. [18] observed a linearly increasing risk for myocardial infarctions of 6.4% per Gy MHD to the complete heart (95% CI 1.3%–16.0%) in patients treated between 1970 and 2009. For V5Gy and myocardial infarction, the authors identified a statistically significant increased risk for V5Gy ≥ 30% of the complete heart (RR 2.02, 95% CI 1.43–2.85, *P* = .008). In a cohort study of 910 BC patients with RT in 2005–2008 in the Netherlands, 30 major cardiac events occurred [17]. This cohort study observed a 16.5% (95% CI 0.6%–35.0%, *P* = .042) increase of the cumulative incidence per Gy of radiation to the complete heart for major cardiac events [17]. A detailed overview of key characteristics and results of these mentioned studies including our study is presented in the supplementary information (Table A.5).

In contrast to these previous studies on individual dosimetry of RT, we did not observe a dose-dependent increase in risk for cardiac events. The MHD in our study were considerably lower than in Darby et al. [12] and Jacobse et al. [18]. Both used radiation therapy charts to reconstruct doses, which may reduce the reliability of reported doses. Our doses were comparable to van den Bogaard et al. [17], but higher than Lorenzen et al. [34] (Table A.5), who both also based their dosimetry on 3D-CT planning records like our study. Beside these discrepancies in MHD, several other systematic differences to previous studies may have contributed to our dissimilar results, namely differences with respect to the treatment period, the definition of the endpoint, and age restriction.

Most previous studies mainly included patients treated before 2000 [12,18,34], while 75% of our patients received RT between 2000 and 2008. Guidelines for RT in BC patients and RT techniques have changed over the years [5]. This led to a reduction of radiation dose to the heart. In addition, clinicians’ awareness of potential cardiac effects due to RT may have increased over the years [35], contributing to treatment selection and reductions of heart dose. Therefore, comparison to earlier studies, which include patients from 1958 to mainly before 2000 is hampered.

We used a broader definition for the cardiac event endpoint compared to earlier studies with a more narrow endpoint, focused on only myocardial infarction [18] or on myocardial infarction, coronary revascularization and death from ischemic heart disease [12,17,34]. In our study, however, a wider range of cardiac morbidities like dysrhythmias or valvular heart disease were included. This might lead to an endpoint that is not significantly associated with heart dose compared to previous studies that used a more specific endpoint. However, as a post-hoc explorative analysis, we conducted separate analyses for the two endpoints cardiac mortality and cardiac morbidity (Table A.6). Despite a small, statistically non-significant increased risk for cardiac mortality associated with DMEAN of the complete heart (OR per 1% increase = 1.03, 95% CI 0.91–1.15, *P* = .66), the results were consistent with the results from the main endpoint of cardiac events.

In our study, 28% of patients were >70 years at time of diagnosis. In comparison, previous studies restricted age at diagnosis to <70 [12], <71 [18], or ≤75 [12,34]. A large cross-sectional study in Germany showed that the 12-month prevalence for myocardial infarction, chronic consequences of myocardial infarction or angina pectoris substantially increases with age in women from 3.4% (55–64 years) to 16.0% (≥75 years) [36]. Therefore, the effect of RT on cardiac events might be diminished by inclusion of a substantial number of women aged 70 and older at time of diagnosis, who are at higher risk for a cardiac event due to their age regardless of differences in radiation dose due to RT.

### 4.3 Strength and limitations

Our study included a large number of cases and controls treated with contemporary 3D-conformal RT between 1998 and 2008 with extensive clinical documentation, including information on cardiac comorbidities at baseline. Evidence on cardiac risk based on individual dosimetry and comprehensive follow-up of patients from this treatment period is sparse internationally and completely missing for Germany so far, making our study an important contribution.

Despite these strengths, our study had limitations. Individual doses were only available for a subset of cases and controls since the heart was not completely visible in some CT scans, preventing heart dosimetry. In addition, for some patients, electronic treatment planning records were not accessible. Therefore, we performed dose predictions to estimate heart dose for cases and controls without individual dosimetry. Nevertheless, sensitivity analyses restricted to cases and controls with individual dosimetry did not reveal differences in risk estimates compared to the main analyses that included dose predictions. Furthermore, information on cardiac morbidity were derived from a self-administered questionnaire. This implies a risk for selection bias. Survivors with a healthy lifestyle might have been more motivated to participate in a questionnaire on late cardiac effects compared to non-responders. Additionally, self-reported events may be prone to information bias through restrictions in memory, misunderstanding of medical diagnoses, or selective reporting [37]. This could potentially result in misclassification. However, a validation study on a patient sample of our study demonstrated a moderate to fair agreement between self-reported events compared to medical records from general practitioners [38].

## 5 Conclusion

Our results provide no evidence that the radiation dose to the heart from contemporary 3D-conformal RT for female breast cancer patients treated between 1998 and 2008 is associated with cardiac events.

## Funding information

This work was funded by the German 10.13039/501100002347Federal Ministry of Education and Research (BMBF). Contract number 02NUK048. The study sponsor had no involvement in the study design, in the collection, analysis and interpretation of data, in the writing of the manuscript, or in the decision to submit the manuscript for publication. This work is part of the PhD-thesis of Dan Baaken at the University Medical Center Mainz.

## Ethical approval

The ESCaRa Study has been approved by the Ethics Committee of Rhineland-Palatinate, Mainz and the Ethics Committee of the University of Ulm. The data protection officer approved using the patients’ hospital records and the performing of a mortality follow-up for the entire cohort without written informed consent of the patients. For the questionnaire survey, individual informed consent was obtained.

## Availability of data

Raw data compliant with the institutional confidentiality policies can be available upon request from the corresponding author.

## Declaration of competing interest

The authors declare that they have no known competing financial interests or personal relationships that could have appeared to influence the work reported in this paper.
