# Supplementary material for: A nested case-control study on radiation dose-response for cardiac events in breast cancer patients in Germany
Source: Breast. 2022 Jun 9;65:1–7. doi: 10.1016/j.breast.2022.05.007 (PMC9207715; doi:10.1016/j.breast.2022.05.007)
Supplement: Multimedia component 1 [file mmc1.docx]

**Appendix A Supplementary information**

**Table A.1 Characteristics of 91 cases and 182 matched controls after breast cancer therapy in 1998–2008 with available doses from the dosimetry sample**

| **Characteristics** | **Cases** | | **Controls** | |
| --- | --- | --- | --- | --- |
|  | *N* = 91 | 100 (%†) | *N* = 182 | 100 (%†) |
| Year of breast cancer diagnosis |  |  |  |  |
| 1998–2000 | 22 | 24.18 | 47 | 25.82 |
| 2001–2003 | 22 | 24.18 | 46 | 25.27 |
| 2004–2006 | 27 | 29.67 | 59 | 32.42 |
| 2007–2008 | 20 | 21.98 | 30 | 16.48 |
| Age at breast cancer diagnosis |  |  |  |  |
| Mean | 64.09 | / | 64.07 | / |
| SD* | 11.23 | / | 11.15 | / |
| Laterality |  |  |  |  |
| Left | 55 | 60.44 | 101 | 55.49 |
| Right | 36 | 39.56 | 81 | 44.51 |
| T-stage |  |  |  |  |
| 1 | 56 | 61.54 | 99 | 54.40 |
| 2 | 23 | 25.27 | 58 | 31.87 |
| 3 | 3 | 3.30 | 4 | 2.20 |
| 4 | 5 | 5.49 | 10 | 5.49 |
| In situ | 3 | 3.30 | 9 | 4.95 |
| Unknown | 1 | 1.10 | 2 | 1.10 |
| N-stage |  |  |  |  |
| 0 | 63 | 69.23 | 109 | 59.89 |
| 1 | 22 | 24.18 | 54 | 29.67 |
| 2 | 2 | 2.20 | 5 | 3.30 |
| 3 | 2 | 2.20 | 7 | 3.85 |
| X | 2 | 2.20 | 6 | 3.30 |
| BMI** |  |  |  |  |
| <25.0 | 35 | 38.46 | 86 | 47.25 |
| ≥25.0 | 49 | 53.85 | 89 | 48.90 |
| Unknown | 7 | 7.69 | 7 | 3.85 |
| History of cardiac disease^‡^ |  |  |  |  |
| Yes | 35 | 38.46 | 70 | 38.46 |
| No/unknown | 56 | 61.54 | 112 | 61.54 |
| Chemotherapy |  |  |  |  |
| Yes | 39 | 42.86 | 74 | 40.66 |
| No | 52 | 57.14 | 102 | 56.04 |
| Unknown | 0 | 0.00 | 6 | 3.30 |
| Endocrine therapy |  |  |  |  |
| Yes | 69 | 75.82 | 120 | 65.93 |
| No | 18 | 19.78 | 43 | 23.62 |
| Unknown | 4 | 4.40 | 19 | 10.44 |
| Type of surgery |  |  |  |  |
| None^§^ | 0 | 0.00 | 1 | 0.55 |
| Breast conserving | 85 | 93.41 | 157 | 86.26 |
| Mastectomy | 6 | 6.59 | 24 | 13.19 |
| Unknown | 0 | 0.00 | 0 | 0.00 |

* SD: Standard deviation ** BMI: Body Mass Index

† Percentages may not add up to a total of 100 due to rounding.

‡ History of cardiac disease at the time of breast cancer diagnosis, including history of cardiac infarction, coronary heart disease, angina pectoris, NYHA≥3, dysrhythmia, vitium cordis or use of a pacemaker.

§ Patients who did not receive breast conserving surgery or mastectomy during their breast cancer therapy.

**Table A.2**

**Mean, median and standard deviations for the complete heart and left anterior heart wall for different exposure metrics for cases and matched controls after breast cancer therapy in 1998–2008 stratified by laterality based on 91 cases and 182 controls with available doses from the dosimetry sample**

| **Structure** | **Metric** | **Laterality** | **Mean** | | **Median** | | **SD** | | **Range** | |
| --- | --- | --- | --- | --- | --- | --- | --- | --- | --- | --- |
|  |  |  | Cases | Controls | Cases | Controls | Cases | Controls | Cases | Controls |
| Complete heart | DMEAN [Gy] | Left | 3.92 | 4.38 | 3.30 | 3.89 | 2.13 | 2.70 | 1.21–10.75 | 1.00–12.85 |
|  |  | Right | 1.59 | 1.70 | 1.47 | 1.46 | 0.73 | 1.15 | 0.54–3.77 | 0.56–9.79 |
|  | V5Gy [Gy] | Left | 12.28 | 13.31 | 9.93 | 11.32 | 9.39 | 12.35 | 0.28–37.99 | 0–84.25 |
|  |  | Right | 2.61 | 3.12 | 0 | 0 | 5.12 | 7.29 | 0–19.89 | 0–45.52 |
| Left anterior heart wall | DMEAN [Gy] | Left | 13.76 | 15.16 | 11.70 | 13.29 | 8.83 | 9.95 | 2.37–34.99 | 0.96–40.85 |
|  |  | Right | 1.35 | 1.56 | 1.20 | 1.29 | 0.63 | 1.06 | 0.23–3.39 | 0.30–7.18 |
|  | V5Gy [Gy] | Left | 57.26 | 55.64 | 57.34 | 61.94 | 25.77 | 30.58 | 3.28–95.36 | 0–100. |
|  |  | Right | 0.59 | 1.89 | 0 | 0 | 2.07 | 7.11 | 0–10.37 | 0–47.02 |

**Figure A.1**

**Boxplots of estimated mean heart dose and V5Gy of cases and matched controls after breast cancer therapy in 1998–2008 depending on case-control status and laterality for the complete heart and the left anterior heart wall based on 91 cases and 182 controls with available doses from the dosimetry sample**


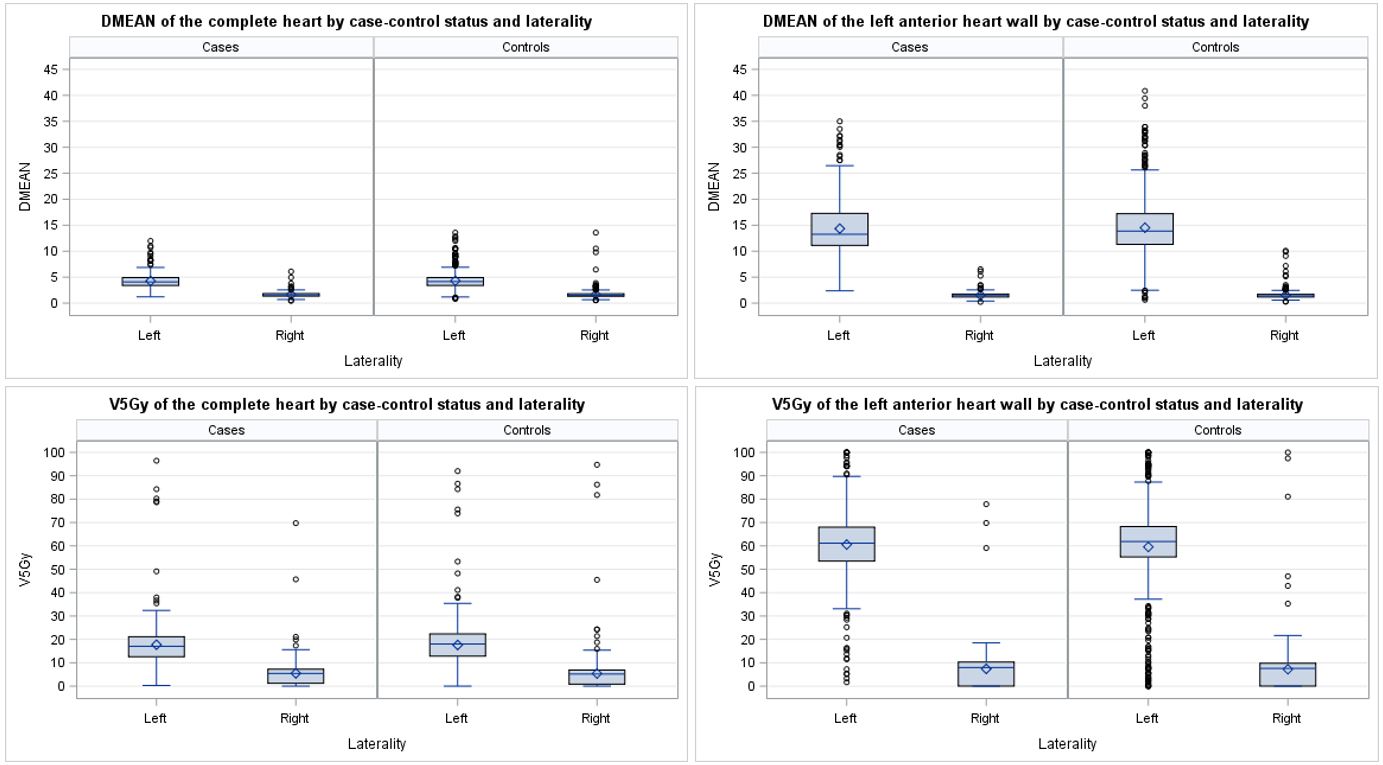


The lines in the boxplots show the median and the rhombs show the mean.

**Table A.3**

**Conditional logistic regression analyses of potential risk factors associated with cardiac morbidity and cardiac mortality of 91 cases and 182 controls after breast cancer therapy in 1998–2008 with available doses from the dosimetry sample**

|  | **Crude** | | | **Adjusted**† | | |
| --- | --- | --- | --- | --- | --- | --- |
| **Variables** | Odds Ratio | 95% CI | *P* | Odds Ratio | 95% CI | *P* |
| Dose groups for complete heart using DMEAN [Gy] |  |  |  |  |  |  |
| 1^st^ quintile (<1.25 Gy) | 1.00* |  |  | 1.00* |  |  |
| 2^nd^ quintile (≥1.25 Gy - <1.79 Gy) | 0.96 | 0.41–2.25 | .92 | 0.89 | 0.37–2.13 | .79 |
| 3^rd^ quintile (≥ 1.79 Gy - <2.71 Gy) | 1.27 | 0.58–2.79 | .56 | 1.25 | 0.56–2.79 | .58 |
| 4^th^ quintile (≥2.71 Gy - <4.73 Gy) | 1.14 | 0.50–2.61 | .75 | 1.07 | 0.46–2.47 | .88 |
| 5^th^ quintile (≥4.73 Gy) | 1.07 | 0.47–2.42 | .88 | 1.05 | 0.46–2.40 | .91 |
| Complete heart using DMEAN [Gy], continuous per 1 Gy | 0.96 | 0.86–1.08 | .52 | 0.96 | 0.86–1.08 | .54 |
| Dose groups for left anterior heart wall using DMEAN [Gy] |  |  |  |  |  |  |
| 1^st^ quintile (<1.15 Gy) | 1.00* |  |  | 1.00* |  |  |
| 2^nd^ quintile (≥1.15 Gy - <2.15 Gy) | 0.92 | 0.38–2.22 | .85 | 0.91 | 0.38–2.21 | .84 |
| 3^rd^ quintile (≥2.15 Gy - <7.56 Gy) | 1.03 | 0.45–2.35 | .95 | 1.06 | 0.45–2.49 | .88 |
| 4^th^ quintile (≥7.56 Gy - <18.07 Gy) | 1.70 | 0.79–3.68 | .17 | 1.63 | 0.75–3.53 | .22 |
| 5^th^ quintile (≥18.07 Gy) | 0.70 | 0.29–1.68 | .42 | 0.71 | 0.29–1.71 | .44 |
| Left anterior heart wall using DMEAN [Gy], continuous per 1 Gy | 0.997 | 0.97–1.03 | .83 | 0.997 | 0.97–1.03 | .85 |
| Chemotherapy |  |  |  |  |  |  |
| No | 1.00* |  |  | 1.00* |  |  |
| Yes | 1.06 | 0.62–1.81 | .83 | 1.12 | 0.64–1.97 | .69 |
| Endocrine therapy |  |  |  |  |  |  |
| No | 1.00* |  |  | 1.00* |  |  |
| Yes | 1.37 | 0.75–2.51 | .31 | 1.38 | 0.74–2.59 | .32 |
| BMI |  |  |  |  |  |  |
| <25.0 | 1.00* |  |  | 1.00* |  |  |
| ≥25.0 | 1.39 | 0.81–2.38 | .23 | 1.36 | 0.79–2.34 | .26 |

* Reference category.** BMI: Body Mass Index
† Radiation-related factors are adjusted for chemotherapy, endocrine therapy and BMI. Chemotherapy, endocrine therapy and BMI are adjusted for each other and DMEAN of the complete heart as continuous variable.

**Table A.4**

**Conditional logistic regression analyses of percentage of heart volume receiving >5Gy and cardiac morbidity and cardiac mortality of 91 cases and 182 controls after breast cancer therapy in 1998–2008 with available doses from the dosimetry sample**

|  | **Crude** | | | **Adjusted**† | | |
| --- | --- | --- | --- | --- | --- | --- |
| **Variables** | Odds Ratio | 95% CI | *P* | Odds Ratio | 95% CI | *P* |
| Groups of percentage of the complete heart receiving >5 Gy [V5Gy] |  |  |  |  |  |  |
| <10% | 1.00* |  |  | 1.00* |  |  |
| 10%-29% | 0.94 | 0.54–1.63 | .82 | 0.94 | 0.54–1.66 | .83 |
| ≥30% | 0.87 | 0.26–2.87 | .82 | 0.82 | 0.24–2.80 | .76 |
| Percentage of the complete heart receiving >5 Gy [V5Gy], continuous per 1% | 0.997 | 0.97–1.02 | .81 | 0.998 | 0.97–1.02 | .89 |
| Groups of percentage of the left anterior heart wall receiving >5 Gy [V5Gy] |  |  |  |  |  |  |
| <10% | 1.00* |  |  | 1.00* |  |  |
| 10%-29% | 0.79 | 0.30–2.07 | .62 | 0.79 | 0.29–2.10 | .63 |
| ≥30% | 1.43 | 0.83–2.46 | .20 | 1.43 | 0.83–2.48 | .20 |
| Percentage of the left anterior heart wall receiving >5 Gy [V5Gy], continuous per 1 % | 1.003 | 0.96–1.01 | .47 | 1.003 | 0.996–1.01 | .47 |

* Reference category.
† Adjusted for chemotherapy, endocrine therapy and BMI.

**Table A.5**

**Characteristics and results of case-control studies and cohort studies on female breast cancer patients with individual dosimetry of radiotherapy and risk for major cardiac events**

| **Study characteristics** | **Darby et al., 2013** | **Van den Bogaard et al., 2017** | **Jacobse et al., 2019** | **Lorenzen et al., 2020** | **ESCaRa, 2021** |
| --- | --- | --- | --- | --- | --- |
| **Country** | Sweden, Denmark | Netherlands | Netherlands | Denmark | Germany |
| **Study type** | Case-control | Cohort | Case-control | Case-control | Case-control |
| **Cases/Controls*** | 963/1205 | 30/910 | 183/182 | 196/413 | 494/988 |
| **Treatment period** | 1958–2001 (Sweden), 1977–2000 (Denmark) | 2005–2008 | 1970–2009 (<10% after 2000) | 1977–2005 | 1998–2008 (>75% >2000) |
| **Mean heart dose** | Mean: Cases: 5.4 Gy  Controls: 4.5 Gy  Left: 6.6 Gy Right: 2.9 Gy | Median: Left: 4.44 Gy Right: 1.31 Gy | Median:  Cases: 8.9 Gy Controls: 8.5 Gy | Median: Cases: 0.84 Gy Controls: 0.71 Gy Left: 2.41 Gy Right: 0.68 Gy | Median: Cases: 3.05 Gy  Controls: 3.11 Gy  Left: 4.30 Gy Right: 1.65 Gy |
| **Endpoint** | Myocardial infarction (I21–I24) Coronary revascularization Death from ischemic heart disease (I20–I25) | Myocardial infarction (I21–I24) Coronary revascularization Death from ischemic heart disease (I20–I25) | Myocardial infarction | Myocardial infarction (I21–I24) Coronary revascularization Death from ischemic heart disease (I20–I25) | Cardiac morbidity: Myocardial infarction, Angina pectoris, Congestive heart failure, Dysrhythmias, Valvular heart disease Cardiac mortality: Cardiac infarction (I21–I23), Chronic heart disease (I25.0–25.9), Acute ischemic heart disease (I21.0–24.9), Congestive heart failure (I50.0–I50.9), Angina pectoris (I20.0–I20.9), Cardiac arrest (I46), Dysrhythmias and conductive disorder (I44.0–I49.9), Vitium cordis (I34.0–37.9) |
| **Inclusion criteria** | Histologically confirmed breast cancer | Surgery Stage I-III invasive carcinoma or CIS | Stage I-III invasive carcinoma or CIS Surgery | Early stage breast cancer,  Surgery | Histologically confirmed primary and locoregional breast cancer (invasive carcinoma or CIS) |
| **Exclusion criteria** | Bilateral breast cancer, Metastatic disease at time of breast cancer diagnosis, Prior cancer (apart from non-melanoma skin cancer) Prior radiotherapy to thoracic area | History of other malignant diseases Prior radiotherapy or chemotherapy before breast cancer | Prior cancer (apart from non-melanoma skin cancer) BC recurrence, Distant metastasis, Second cancer, Heart failure or valvular disease at MI diagnosis | Bilateral breast cancer, Unknown laterality, Prior cancer (apart from non-melanoma skin cancer)  No radiotherapy for breast cancer | Bilateral breast cancer, Primary metastatic disease  No radiotherapy for breast cancer |
| **Age restriction** | <70 years at diagnosis (Sweden), <75 years at diagnosis (Denmark) | No age restriction | <71 years at diagnosis | ≤75 years at diagnosis | No age restriction (28% >70 at diagnosis) |
| **Matching variables** | Country of residence, Age and year of breast cancer diagnosis (both within 5 years),  Receipt of radiotherapy,  No recurrence of breast cancer, other cancer or major coronary event before index date | n.a. | Age and year of breast cancer diagnosis (both within 5 years) | Age and year of breast cancer diagnosis (both within 5 years), Time since radiotherapy (within 5 years) | Age and year of breast cancer diagnosis (both within 5 years), Study center, cardiac comorbidities |
| **Latency time** | No | No | No | No | 1 year after breast cancer diagnosis |
| **Age at diagnosis** | Not available | Median: 59 years | Median Cases 50.2 years Controls 20.5 years | Median 62.5–63.6 years | Mean Cases 64.09 years  Controls 64.07 years |
| **Median follow-up** | Not available | 7.6 years | Cases 13.6 years Controls: 13.7 years | 7.3–7.5 years | Cases: 12.52 years Controls: 12.76 years |
| **Definition of baseline cardiac risk** | Prior of ischemic heart disease, Risk factors other than ischemic heart disease: Circulatory disease, COPD, Smoking, BMI, Analgesic medication, Other medications (hormone replacement, thyroid, other) | Prior ischemic heart disease, Risk factors other than ischemic heart disease: circulatory disease, COPD, smoking, BMI, analgesic medication, other medications (hormone replacement, thyroid, other) | Prior cardiovascular diseases, Diabetes; Hypertension, Smoking, BMI | Prior myocardial infarction or angina pectoris, Prior circulatory diseases, Hypertension, Diabetes, COPD, Smoking, Obesity, Hormone replacement | Prior cardiac infarction, Coronary heart disease, Angina pectoris, NYHA ≥3, Dysrhythmia, Vitium cordis, Pacemaker |
| **Occurrence of baseline cardiac risk** | Cases: 63.3% Controls: 50.2% | More than one risk factor: 57.5% | Cases: 57.9%  Controls: 44.5% | Prior myocardial infarction or angina pectoris: Cases 13.8% Controls: 2.4%  Prior circulatory disease:  Cases: 17.9% Controls: 14.5% | Cases: 17% Controls: 17% |
| **Risk per Gy** | 7.4% increase per Gy (95% CI 2.9%–14.5%, *P* <0.001) | 16.5% increase in cumulative incidence per Gy (95% CI 0.6%–35.0%, *P* 0.042) | 6.4% increase per Gy (95% CI 1.3–16.0) | 19% increase per Gy (95% CI 1%–63%, *P* 0.02) | OR 0.99 (95% CI 0.94–1.05, *P* 0.73) |

* For cohort studies events and cohort size is reported

**Table A.6**

**Conditional logistic regression analysis of risk for cardiac mortality and cardiac morbidity for exposure of the complete heart and exposure of the left anterior heart wall due to radiotherapy in female breast cancer patients treated between 1998-2008**

|  | **Cardiac mortality*** | | | **Cardiac morbidity*** | | |
| --- | --- | --- | --- | --- | --- | --- |
| **Variables** | Odds Ratio† | 95% CI | *P* | Odds Ratio† | 95% CI | *P* |
| Complete heart using DMEAN [Gy], continuous per 1 Gy | 1.03 | 0.91-1.15 | .66 | 0.98 | 0.92-1.04 | .47 |
| Percentage of the complete heart receiving >5 Gy [V5Gy], continuous per 1% | 1.00 | 0.98-1.02 | .86 | 1.00 | 0.99-1.01 | .78 |
| Left anterior heart wall using DMEAN [Gy], continuous per 1 Gy | 1.00 | 0.97-1.04 | .80 | 1.00 | 0.99-1.01 | .78 |
| Percentage of the left anterior heart wall receiving >5 Gy [V5Gy], continuous per 1 % | 1.00 | 0.99-1.01 | .89 | 1.00 | 0.996-1.01 | .86 |

* Analysis for cardiac mortality based on 105 cases and 210 controls; analysis of cardiac morbidity based on 389 cases and 778 controls
† Adjusted for chemotherapy, endocrine therapy and BMI.
